# Association between hand grip strength and exercise addiction: differences by sport category and sex among elite athletes

**DOI:** 10.3389/fpsyg.2025.1597239

**Published:** 2025-07-21

**Authors:** Barış Karaoğlu, Ebru Ceviz, Şaban Ünver, İsa Çiftçi, Gönül T. Demir, Burcu Güvendi, Celal Bulgay, Merve Alpay, Ferenc Ihasz, Alföldi Zoltan, Rita Kovacsik, Angéla Somogyi, Attila Szabo

**Affiliations:** 1 Sports Science Faculty, Bingol University, Bingol, Türkiye; 2 Yaşar Doğu Faculty of Sports Science, Ondokuz Mayıs University, Samsun, Türkiye; 3 Independent Researcher, Ankara, Türkiye; 4 Sports Science Faculty, Yalova University, Yalova, Türkiye; 5 Physical Education and Sports, Institute of Health Sciences, Sivas Cumhuriyet University, Sivas, Türkiye; 6 Faculty of Health and Sport Sciences, Széchenyi István University, Győr, Hungary

**Keywords:** athletes, athletic performance, exercise addiction, hand grip strength, sports

## Abstract

**Introduction:**

Handgrip strength (HGS) is a key indicator of upper-body strength and overall physical fitness. While its links to health and sports performance have been widely studied, its relationship with the risk of exercise addiction (REA) remains unclear. Thus, the present study examines the relationship between HGS and REA across various sports disciplines, sexes, and national contexts, aiming to deepen our understanding of their intricate interplay.

**Methods:**

Using a cross-sectional research design, data were collected from 1,211 high-level athletes from Türkiye (*n* = 656) and Hungary (*n* = 555), spanning a wide range of competitive sports. The measures involved demographic questions, the Exercise Addiction Inventory to assess REA, and HGS assessments.

**Results:**

We found significant negative correlations between HGS and REA among Turkish female athletes competing in racquet and water sports (*p* < 0.05). In contrast, significant positive correlations emerged in team sports played with hands and target-based sports (*p* < 0.05). Statistically significant HGS-REA correlations were also observed among Turkish male athletes participating in gymnastics and esthetic sports (*p* < 0.05). Among Hungarian athletes, a significant HGS-REA correlation was found in male racquet sports athletes and female athletes engaged in combat sports (*p* < 0.05). Regardless of sports discipline, a statistically significant positive correlation was observed exclusively among Turkish female athletes (*p* < 0.05).

**Conclusion:**

The present study reveals that the correlations between HGS and the risk of REA may vary depending on country, sex, and type of sport. The findings indicate that HGS may be either positively or negatively associated with REA in specific sports disciplines.

## 1 Introduction

Physical exercise is a structured, systematic, and repetitive activity designed to maintain and/or enhance physical fitness and overall health (Bruno et al., 2014; Caspersen et al., 1985). Its positive impact on human health has frequently been acknowledged from ancient times to the present day and supported by extensive scientific evidence (Paffenbarger et al., 2001; Ruegsegger and Booth, 2018). Recent studies further emphasize that regular physical activity plays a fundamental role in maintaining fitness and significantly contributes to the prevention and management of chronic diseases, ultimately improving quality of life (Marquez et al., 2020; Ruegsegger and Booth, 2018). In addition to its physiological benefits, exercise interventions are increasingly recognized as effective therapeutic strategies for managing mental health disorders such as depression, anxiety, and stress (Kandola and Stubbs, 2020; Malchow et al., 2013; Sicilia et al., 2023). Moreover, group exercise programs may boost social interaction and augment mental well-being through physical and biochemical mechanisms (Mandolesi et al., 2018).

While exercise provides substantial physical and psychological benefits, excessive and unregulated participation can have adverse health effects (Lichtenstein et al., 2014; Weinstein and Szabo, 2023). When exercise shifts from health-promoting behavior to compulsive and obsessive training, there is a risk of exercise addiction (REA) (Vansteene et al., 2022). The REA is characterized by loss of control, compulsive exercise patterns, and adverse personal, social, and professional consequences (Ordu, 2022; Szabo et al., 2015). Additionally, frequent urges to exercise, exhibiting aggression or anxiety when unable to engage in physical activity, and a tendency to exercise for longer durations than intended are indicators of REA (Demir and Türkeli, 2019).

Exercise addiction (EA) is divided into two subtypes: primary and secondary. Primary EA happens when exercise itself becomes the focus of addiction, which is often seen in elite athletes aiming for peak performance. On the other hand, secondary EA is connected to psychological issues such as eating disorders, distorted body image, where excessive exercise is used to handle a different goal related to weight control or body image concerns (De Coverley Veale, 1987; Freimuth et al., 2011; Szabo and Demetrovics, 2022). Despite sharing neurobiological similarities with substance addiction (Leeman and Potenza, 2012), EA has not yet been officially recognized as a psychiatric disorder. The Diagnostic and Statistical Manual of Mental Disorders (DSM-5) and the International Classification of Diseases (ICD-11) do not list REP as a clinical condition, emphasizing the need for more research to confirm its validity (American Psychiatric Association, 2013; World Health Organization, 2019).

Research on REA has significantly evolved since its conceptual emergence in the 1970s, experiencing substantial growth between the 1980s and 2000s and reaching its peak in recent years (Adams and Kirkby, 1998; Berczik et al., 2012; Blaydon and Lindner, 2002; Çetin et al., 2021; Godoy-Izquierdo et al., 2023; Krivoschekov and Lushnikov, 2011; Leeman and Potenza, 2012; Demir, 2022; Weinstein and Weinstein, 2014). However, findings on its prevalence remain inconsistent, mainly due to methodological differences, variations in assessment tools, and differences between theoretical models (Godoy-Izquierdo et al., 2023; Gonçalves et al., 2019; Szabo et al., 2015; Turton et al., 2017). This inconsistency underscores the need for reliable and generalizable assessment tools to evaluate REA accurately. In parallel with these measurement challenges, researchers assessing physical performance in relation to EA also face practical barriers. One of the key challenges in measuring physical performance is the high cost of equipment, the need for specialized facilities, and the requirement for technical expertise. Researchers have increasingly adopted low-cost, accessible, and practical alternatives, among which hand grip strength (HGS) testing has gained prominence (Kim et al., 2022).

As a significant marker of physical fitness, in adult and older general populations, HGS demonstrates strong correlations with aerobic capacity, physical activity levels, functional capacity, and walking speed, lower limb strength, and overall athletic performance; its association with various physiological parameters highlights its importance in assessing functional capacity and fitness level (Bohannon, 1998; Braun et al., 2018; Cooper et al., 2010; Dag et al., 2021; Desrosiers et al., 1997; Kuh et al., 2005; Vaıdya and Narıya, 2021). Beyond its role in sports performance, HGS is associated with critical health markers, including metabolic disorders (Byeon et al., 2019; Merchant et al., 2020), sarcopenia (Marques et al., 2019), cardiovascular function (Zhu et al., 2020), cognitive impairments (Zammit et al., 2019), mental health (Cabanas-Sánchez et al., 2022), and sarcopenia (Pratt et al., 2021). A recent review further demonstrated that low HGS was linked to cardiovascular diseases, sarcopenia, and reduced quality of life (Vaishya et al., 2024).

Although the REA has been extensively studied regarding its mental and physiological consequences, its association with objective physical strength measures remains unexplored. Characterized by excessive and compulsive exercise behaviors, REA has been linked to various health outcomes, yet its relationship with HGS and other physical performance indicators has received limited attention. Investigating this relationship holds significant potential for elucidating the mechanisms through which compulsive exercise behaviors influence both athletic performance and overall psychological well-being. Considering increasing concerns regarding exercise addiction, particularly among high-performing athletes who consistently exceed their physical limits, addressing this critical gap in the literature is imperative. Furthermore, the current body of research remains limited in its examination of variations across sport disciplines, sexes, and demographic groups, thereby constraining a comprehensive understanding of the complex interaction between psychological and physiological factors in athletic contexts. This line of inquiry aims to advance a more nuanced and integrative perspective on the role of exercise addiction in shaping athlete health and performance outcomes. Grounded in Self-Determination Theory (SDT), this study explores how imbalances in psychological needs, competence, autonomy, and relatedness may contribute to exercise addiction, ultimately influencing physical strength and exercise behaviors (Deci and Ryan, 2000).

The SDT emphasizes intrinsic motivation, autonomy, competence, and relatedness in shaping human behavior and well-being. In the context of HGS and REA, *autonomy* is crucial in determining an athlete’s approach to training and recovery. Athletes with high autonomy are more likely to engage in regular exercise routines, which may foster high levels of HGS without leading to addiction. *Competence*, fueling mastery and effectiveness, is closely linked to physical performance. Athletes with higher levels of HGS often experience greater feelings of competence, which can enhance motivation. However, when this drive for competence becomes excessive, it may increase the REA as athletes push themselves beyond healthy physical limits to maintain or improve performance, as highlighted by the interactional model of exercise addiction (Dinardi et al., 2021). Lastly, *relatedness*, or the need to feel connected with others, can influence an athlete’s approach to exercise. Strong social connections and community support may mitigate the REA by fostering a healthier, more balanced attitude toward exercise. SDT provides a valuable framework to understand how psychological needs affect physical strength and the potential for exercise addiction, highlighting the interplay between motivation, performance, and well-being in athletes.

Consequently, the present study examines the association between HGS and REA among elite athletes from diverse sporting disciplines and both sexes in Türkiye, which is a collectivist nation, where REA prevalence has been reported at a striking 18% and Hungary, an individualist nation, which demonstrates a considerably lower prevalence of 7.5% (Chhabra et al., 2024). By employing a dual-national and multi-sport design, this research aims to deepen the scientific understanding of REA through a comparative lens, while considering the differences between individualist and collectivist cultures (Chhabra et al., 2024). Grounded in SDT, the study explores potential links between HGS and REA across athletic domains.

Although the exploratory nature of the research precluded the formulation of specific hypotheses, it was anticipated that notable cross-cultural differences would emerge, thereby shedding light on the sociocultural and physiological factors that may contribute to REA. While the primary aim of the present study is not to elucidate the psychological or physiological mechanisms underlying REA directly, it seeks to establish empirical associations between physical performance indicators and behavioral tendencies related to exercise. By identifying reliable, low-cost markers, such as HGS, that may correlate with REA tendencies, this research lays the groundwork for future studies to explore the biopsychosocial mechanisms driving compulsive exercise behavior.

## 2 Materials and methods

### 2.1 Ethical approval

The present study was ethically approved by the Ethics Committee of Bingöl University (Approval no: 24/17, dated October 24, 2024) and the Ethics Committee of Széchenyi István University, Győr (Approval no: SZE ETT-152024 (X.15), dated October 15, 2024). The study adhered to the principles of the Declaration of Helsinki (World Medical Association, 2013) and followed ethical standards specific to the sports and exercise sciences. All participating athletes provided written and verbal informed consent before participating in the study. They also consented to the anonymous publication of the results (i.e., only group-based).

### 2.2 Study model

The present study employed a cross-sectional design, in which data were collected at a single point in time. Cross-sectional studies aim to assess various characteristics, including participants’ perspectives, interests, skills, abilities, and attitudes regarding a specific subject or event. Typically, these studies require larger sample sizes compared to other research designs (Karasar, 2017).

### 2.3 Sample size estimation

A power analysis was conducted using G*Power 3.1 software to determine the required sample size. The effect size criteria proposed by Cohen (1988) were utilized for this analysis. According to these criteria, an effect size of 0.10 indicates a small effect, 0.30 represents a medium effect, and 0.50 corresponds to a large effect. Given the scope of the present study, a small effect size (0.10) was chosen to ensure an adequately large sample size. The power analysis was conducted with 99% statistical power and a 5% error margin (two-tailed) using the Exact Correlation: Bivariate regular model. This analysis indicated that a minimum of 195 athletes per group was required, leading to a total sample size of 390 athletes for the study. Our sample size exceeded the minimum.

### 2.4 Participants

Participant inclusion criteria: The present study included elite athletes who had participated in national and international competitions. Engage in regular training, voluntarily agree to take part in the study, have consistently continued their training over the past 3 months, and have not experienced any serious health problems during this period.

Participant exclusion criteria included: Individuals with musculoskeletal disorders or neurological conditions, those who had sustained an injury within the past 3 months, participants who provided incomplete or inaccurate questionnaire responses, or were unable to complete the measurements. Additionally, those who did not train regularly for at least 3 months were also excluded from the study. The first study involved 656 athletes from Türkiye. Specifically, 184 of the participants were female (mean age 19.16 ± 4.19 years; height 165.73 ± 6.26 cm; weight 58.97 ± 10.56 kg; sport experience 8.36 ± 3.24 years; HGS dominant 32.10 ± 9.67 kg; REA 19.89 ± 3.61 total) and 472 were male athletes (mean age 22.09 ± 6.83 years; height 177.09 ± 7.84 cm; weight 75.94 ± 13.96 kg; sport experience 10.48 ± 4.64 years; HGS dominant 47.06 ± 10.06 kg; REA 20.12 ± 3.32 total).

The second study involved 555 athletes from Hungary. Of the participants, 181 were female (mean age 22.27 ± 5.57 years; height 168.38 ± 8.00 cm; weight 62.27 ± 11.24 kg; sports experience 12.24 ± 5.34 years; HGS dominant 34.96 ± 8.10 kg; REA 19.86 ± 3.41 total) and 374 were male athletes (mean age 22.74 ± 5.65 years; height 180.92 ± 7.88 cm; weight 79.86 ± 14.75 kg; sport experience 12.07 ± 5.50 years; HGS dominant 49.75 ± 10.77 kg; REA 19.63 ± 3.46 total).

To enhance the precision and effectiveness of the analyses, data were systematically categorized into distinct groups based on the unique dynamics and characteristics of each sports discipline. The present categorization framework was meticulously developed to facilitate analyses that account for the specific requirements, physical demands, and technical aspects associated with each type of sport (Cádiz Gallardo et al., 2023; Cronin et al., 2017; Mitchell et al., 2005; Starzak et al., 2024). The established categories are detailed as follows: Racquet sports (*n* = 102; badminton, squash, tennis, table tennis), Team Sports Played by Hand (*n* = 175; handball, basketball, and volleyball), Team Sports Played by Foot (*n* = 168; football and futsal), Water Sports (*n* = 106; swimming); Combat Sports Focused on Hands (*n* = 160; boxing and wrestling), Combat Sports Combining Hand and Foot (*n* = 158; karate, taekwondo, Muay Thai, Judo, kickboxing, Brazilian Jiu-Jitsu, and Kung Fu), Lower Extremity Equipment-Based Sports (*n* = 80; snowboarding, skiing, and roller skating), Gymnastics and esthetic Sports (*n* = 107; cheerleading, dance, and gymnastics), Power Sports (*n* = 85; fitness, street workout, hammer throw, shot put, and weightlifting), Target-Based Sports (*n* = 70; archery and shooting). Two researchers checked the categorization made with 100% agreement by two other researchers to ensure proper classification.

### 2.5 Data collection tools

#### 2.5.1 Personal information form

To determine the demographic characteristics of the athletes participating in the present study, they were asked questions regarding their sex, age, height, weight, sports experience, sports discipline, and dominant hand usage. All the data were collected in 2025.

#### 2.5.2 Exercise addiction inventory

The Exercise Addiction Inventory (EAI), developed by Terry et al. (2004), is a brief yet psychometrically valid tool for assessing the risk of REA. It consists of six items rated on a 5-point Likert scale from “strongly disagree” to “strongly agree.” Based on the components model of addiction (Griffiths, 2005), the EAI captures the core dimensions of addictive behaviors. Early studies confirmed its reliability and validity, making it a valuable instrument for researchers and practitioners. The Hungarian adaptation of the EAI was conducted in two phases: Demetrovics and Kurimay (2008) oversaw the initial translation and pilot testing, while Szabo (2021) carried out its psychometric validation. Confirmatory factor analysis (CFA) demonstrated a good model fit, and the instrument showed acceptable internal reliability (Cronbach’s *α* = 0.71). The Turkish EAI version, validated by Aydın et al. (2023) with a sample of university students, exhibited strong internal consistency, with omega and alpha coefficients of 0.81 and 0.80, respectively. Significant correlations between the Turkish EAI and the Sports Engagement Scale supported its concurrent validity.

#### 2.5.3 Hand grip strength

Upper extremity muscle strength was evaluated through hand grip strength (HGS) measurements. The assessment used a Smedley-type hand dynamometer (Grip-D 5101; Takei, Niigata, Japan). Athletes held the dynamometer in their tested hands, with their arms extended and positioned at a 15° angle from the body. They were instructed to exert maximum isometric force by squeezing the dynamometer for 5 s. The dynamometer handle was adjusted to align with the second interphalangeal joints of the four fingers and the base of the thumb. Each handgrip strength was measured twice, with the highest value recorded to the nearest 0.1 kg. Measurements were taken for both the dominant and non-dominant hands; however, only the dominant hand results were considered (Kim et al., 2022). The same instrument was used in the Türkiye and Hungarian studies along the same measurement format.

### 2.6 Statistical analysis

The statistical data analysis was performed using Microsoft Excel and SPSS Mac 27.0 software. In the initial stage of the analysis process, missing and erroneous data were assessed to ensure the suitability of the analyses and the fulfillment of assumptions. As a result of this evaluation, data from participants with missing or incorrectly completed responses (for example, providing multiple answers to the same question while completing the EAI) were excluded from the analysis. Analyses were then conducted on data from 1,211 high-level athletes (only three athletes were excluded). To test the normality assumptions, skewness and kurtosis values were examined. In this context, skewness values between −2 and +2, as recommended by George and Mallery (2010), were considered, and it was determined that the data followed a normal distribution. Pearson product–moment correlation coefficients were calculated to examine the relationships between HGS and the total Exercise Addiction Inventory (EAI) scores. Analyses were first conducted separately by sport discipline, country, and sex to explore potential subgroup-specific patterns. In a second step, athletes were grouped by country and sex only to increase statistical power and assess broader trends. Statistical significance was set at *p* < 0.05, and effect sizes were interpreted using standard thresholds for correlation strength (i.e., weak, moderate, strong, and very strong).

## 3 Results

In the initial phase of the present study, an analysis was conducted to examine the relationship between HGS and REA across different sports disciplines in each country. This approach aimed to provide a more detailed and nuanced interpretation of the findings.

The analysis of racquet sports (RS) athletes revealed a statistically significant negative correlation among Turkish female athletes (*p* < 0.05). In contrast, a significant positive correlation was observed among Hungarian male athletes (*p* < 0.05). However, no statistically significant associations were detected among Turkish or Hungarian female athletes (*p* > 0.05; Table 1).

**Table 1:** Hand grip strength association with the risk of exercise addiction within the sports disciplines.

Variable	Sport disciplines	Sex	Country	*N*	Exercise addiction	
					r	*p*
Hand grip strength	RS	Female	Türkiye	7	**−0.799** ^*^	**0.031**
			Hungary	13	0.454	0.119
		Male	Türkiye	49	−0.116	0.427
			Hungary	33	**0.344** ^*^	**0.050**
	TSPH	Female	Türkiye	10	**0.699** ^*^	**0.024**
			Hungary	26	0.192	0.347
		Male	Türkiye	79	0.153	0.180
			Hungary	60	−0.222	0.089
	TSPF	Female	Türkiye	10	−0.241	0.502
			Hungary	17	−0.384	0.128
		Male	Türkiye	77	−0.160	0.165
			Hungary	64	−0.026	0.837
	WS	Female	Türkiye	14	**−0.696** ^*^	**0.006**
			Hungary	19	−0.321	0.194
		Male	Türkiye	37	−0.124	0.466
			Hungary	36	0.087	0.615
	CSFH	Female	Türkiye	27	0.111	0.583
			Hungary	19	**0.627** ^**^	**0.004**
		Male	Türkiye	67	0.102	0.412
			Hungary	47	0.019	0.899
	CSCHF	Female	Türkiye	32	0.109	0.553
			Hungary	26	0.189	0.356
		Male	Türkiye	63	0.156	0.222
			Hungary	37	0.058	0.735
	LEEBS	Female	Türkiye	16	−0.347	0.187
			Hungary	10	0.591	0.072
		Male	Türkiye	25	0.323	0.124
			Hungary	29	−0.099	0.609
	GAS	Female	Türkiye	18	0.134	0.597
			Hungary	49	0.248	0.086
		Male	Türkiye	22	**0.651** ^**^	**0.001**
			Hungary	18	−0.195	0.439
	PS	Female	Türkiye	11	−0.080	0.814
			Hungary	2	---	---
		Male	Türkiye	37	−0.030	0.859
			Hungary	35	−0.274	0.111
	TBS	Female	Türkiye	39	**0.408** ^**^	**0.010**
			Hungary	---	---	---
		Male	Türkiye	16	−0.425	0.101
			Hungary	15	0.249	0.371

^*^p < 0.05; ^**^p < 0.01; RS, Racquet sports; TSPH, Team Sports Played by Hand; TSPF, Team Sports Played by Foot; WS, Water Sports; CSFH, Combat Sports Focused on Hands; CSCHF, Combat Sports Combining Hand and Foot; LEEBS, Lower Extremity Equipment-Based Sports; GAS, Gymnastics and esthetic Sports; PS, Power Sports; TBS, Target-Based Sports. Correlation strength was interpreted using conventional thresholds: r = 0.10–0.29 as weak, 0.30–0.49 as moderate, 0.50–0.69 as strong, and ≥0.70 as very strong (Cohen, 1988). The bold values indicates *p* < 0.05.

In the team sports played by hand (TSPH), a statistically significant positive correlation was identified among Turkish female athletes (*p* < 0.05), whereas no significant relationships were found among Turkish male athletes or Hungarian athletes (*p* > 0.05; Table 1).

In water sports (WS), a statistically significant negative correlation was detected among Turkish female athletes (*p* < 0.05), while no significant associations were observed among Turkish athletes or Hungarian male athletes (*p* > 0.05; Table 1).

In combat sports focused on hands (CSFH), a statistically significant positive correlation was detected among Hungarian female athletes (*p* < 0.05). However, no significant associations were observed among Turkish athletes or Hungarian male athletes (*p* > 0.05; Table 1).

For the gymnastics and esthetic sports (GAS) the analysis indicated a statistically significant positive correlation among Turkish male athletes (*p* < 0.05); however, no significant relationships were identified among Turkish female athletes or Hungarian athletes (*p* > 0.05; Table 1).

In the target-based sports (TBS), a statistically significant positive correlation was found among Turkish female athletes (*p* < 0.05), whereas no significant associations were detected among Turkish male athletes or Hungarian athletes (*p* > 0.05; Table 1).

Furthermore, no statistically significant correlations were observed in team sports played by foot (TSPF), combat sports combining hand and foot (CSCHF), lower extremity equipment-based sports (LEEBS), and power sports (PS) athlete groups (*p* > 0.05; Table 1).

In the second phase of the present study, we opted to combine the participants into a single group to enhance the robustness of the statistical results. The relationship between HGS and REA was examined without differentiation based on sports disciplines, focusing solely on country and sex comparisons. Accordingly, a statistically significant positive correlation was observed among Turkish female athletes (*p* < 0.05). However, no significant associations were found among Turkish male athletes (*p* > 0.05). Similarly, no statistically significant correlations were identified in either male or female Hungarian athletes (*p* > 0.05; Table 2).

**Table 2:** Association analysis of hand grip strength with the risk of exercise addiction among all athletes grouped in a population-based manner.

Variable	Country	Sex	*N*	Exercise addiction	
				r	*p*
Hand grip strength	Türkiye	Female	184	**0.087** ^*^	**0.022**
		Male	472	0.031	0.491
	Hungary	Female	181	0.126	0.091
		Male	374	0.093	0.072

^*^p < 0.05; Correlation strength was interpreted using conventional thresholds: r = 0.10–0.29 as weak, 0.29–0.49 as moderate, 0.50–0.69 as strong, and ≥0.70 as very strong (Cohen, 1988). The bold values indicates *p* < 0.05.

## 4 Discussion

To the best of our knowledge, the present study is the first one to comprehensively examine the relationship between HGS and REA in a large cohort of athletes across various sports disciplines in Türkiye and Hungary. As a widely recognized indicator of upper extremity muscle strength, HGS, in addition to its extensive use in clinical and athletic performance assessments, also appears to be related to REA, probably on motivational grounds, the specifics of which are subject to future theoretically driven empirical research. The results of the present study underscore the importance of understanding how physical factors affect the REA and stress the need for sport-specific and sex-sensitive approaches to studying EA, which aligns with SDT’s focus on individual motivation and behavior.

Expanding on the broader implications of REA, previous research has examined its direct impact on athletic performance. A study on Turkish athletes found that those with high REA levels exhibited lower performance across all track and field disciplines, suggesting REA is a critical factor negatively influencing athletic outcomes (Çetin et al., 2021). Nevertheless, despite its relevance, the validity of REA assessment tools for athletes is controversial. Since athletes undergo prolonged and intense training, elevated exercise levels are likely performance-driven rather than indicative of pathological dependence. A lack of distinction between adaptive high training loads and compulsive exercise behaviors may lead to false-positive REA classifications, misinterpreting high training volume as compulsive behavior (Chapa et al., 2018; Müller et al., 2015; Plateau et al., 2014). Additionally, the absence of universally accepted criteria for excessive exercise makes REA classification challenging. While no clear cut-off exists, researchers largely agree that compulsive exercise, when detrimental to physical and mental health, aligns with addictive behavior (Costa et al., 2015; Kostorz et al., 2022). These inconsistencies highlight the need for standardized and reliable assessment tools. The present study adopted a sport-specific approach to address this methodological gap, assessing each discipline separately to distinguish training demands from performance expectations.

Among various sports disciplines, RS, including badminton, table tennis, tennis, and squash, have garnered increasing scientific interest in recent years (Cádiz Gallardo et al., 2023; Cece et al., 2020; Deng et al., 2023; Lees, 2003; Rigozzi et al., 2023). The present study identified a significant negative correlation between REA and HGS among Turkish female athletes, whereas a positive correlation was observed among Hungarian male athletes. These findings suggest that population- and sex-specific factors may influence the relationship between physical strength and REA. In Turkish female athletes, the negative correlation implies that higher REA levels, potentially associated with compulsive or excessive exercise, may be linked to lower HGS, possibly reflecting the physiological cost of overtraining or a maladaptive exercise pattern. Conversely, the positive correlation observed among Hungarian male athletes suggests that elevated REA levels might correspond to greater HGS, putatively indicating a more adaptive or performance-driven training approach, wherein increased exercise intensity contributes to enhanced physical strength. Therefore, athletes in RS disciplines may develop stronger HGS relative to other sports, which could partly explain the observed correlations between HGS and exercise addiction risk in these groups.

These contrasting findings show the complexity of the REA-HGS relationship, emphasizing the significant roles of population context, sex differences, and sport-specific training regimens in shaping this dynamic. Given the multifaceted nature of REA, no single theoretical model can fully elucidate the underlying mechanisms (Godoy-Izquierdo et al., 2023; Szabo and Demetrovics, 2022). A literature review further highlights the protective role of structured physical activity, as demonstrated by Liu et al. (2019), who found that a 12-week badminton training program significantly reduced internet addiction. However, the influence of sex differences on such relationships warrants further investigation. Existing research indicates distinct physical activity patterns between men and women, indicating the necessity for sex-specific analyses in REA studies (Dumitru et al., 2018). Adopting sex-sensitive approaches and considering the physiological and psychosocial dimensions of REA may contribute valuable insights to the field. Thus, the present study highlights sex and cultural differences when examining the interplay between REA and physical strength, reinforcing the importance of a broader contextual perspective in future research.

In the present study, analysis in WS athletes revealed a statistically significant negative correlation between REA and HGS exclusively among Turkish female athletes, suggesting distinct physiological and psychosocial dynamics. Training regimens, sociocultural influences, and attitudes toward exercise addiction may contribute to this association. Similarly, a negative correlation in female RS athletes suggests that they may share similar physical and psychological power with WS ones. The absence of a similar correlation in other groups underlines that this relationship may be population-specific rather than universal. Notably, the lack of a negative correlation among Hungarian female athletes may reflect differences in training culture and physiological adaptations. In contrast, the absence of significance in male athletes suggests a potential moderating role of sex in the REA-HGS relationship. Elevated REA levels may be linked to compulsive exercise, ultimately impairing performance (Krivoschekov and Lushnikov, 2011). Swimmers demonstrate higher training persistence than other endurance athletes, potentially increasing susceptibility to excessive exercise (da Cruz et al., 2024). However, some evidence suggests swimming may be a protective factor against addictive behaviors (Sunarno et al., 2023). The present findings can inform future research, and studies with larger athlete cohorts may enhance the generalizability of this relationship.

In contrast to racket and water sports, a statistically significant positive correlation between REA and HGS was observed only among Turkish female athletes in the TSPH group, whereas no significant relationships were found among Turkish male or Hungarian athletes. These findings suggest that team sports may impose distinct physical and psychological demands compared to individual sports. The positive correlation in Turkish female athletes may be attributed to team sports’ endurance- and strength-oriented nature, which require greater physical contact, sustained training consistency, and social cohesion. This collective dynamic may enhance motivation and reinforce training adherence, strengthening the REA-HGS relationship. The absence of significance among Hungarian female athletes highlights the need for further research.

Grip strength is essential across all disciplines, particularly for shooting and passing accuracy, with evidence suggesting that athletes with longer fingers and larger hand surface areas exhibit greater HGS, enhancing performance (Visnapuu and Jürimäe, 2007). Among Italian national female basketball players, arm length was identified as a key predictor of grip strength while correlations with height and BMI were weak (Pizzigalli et al., 2017). Beyond physical performance, structured team-based training has been linked to reduced problematic smartphone use (PSU) and improved mental health among university students (Xiao et al., 2021). Despite the extensive literature on problematic exercise, limited research differentiates team and individual sports within the REA framework (Griffiths et al., 2024). By establishing a significant positive REA-HGS relationship among Turkish female athletes, this study enhances understanding of hand-based team sports and their broader psychological implications. Furthermore, the rising international success of Turkish female volleyball players may further shape this association, revealing the need for sport-specific and sex-sensitive analyses in REA research.

Another domain in which Turkish athletes have demonstrated an increasing success rate in global rankings is TBS. In the present study, a statistically significant positive correlation was identified among Turkish female athletes, whereas no significant association was observed among Turkish male or Hungarian athletes. The absence of a significant correlation between Turkish male and Hungarian athletes suggests that this relationship may not be universal but rather influenced by population- and sex-specific differences. This discrepancy could be attributed to variations in cultural, physiological, and training-related factors. Furthermore, the lack of a similar correlation among Turkish male athletes may be explained by the absence of highly elite-level male competitors in the analysis, representing a key limitation of the present study. To obtain more robust and generalizable findings, future research should incorporate elite athletes and classify participants according to their athletic achievement levels (sub-elite, elite, and highly elite) (Kazan et al., 2024). Adopting this approach would facilitate a more comprehensive examination of the relationship between performance metrics and REA in TBS by providing deeper insights into the variability observed across different populations.

A statistically significant positive correlation between REA and HGS was observed among Hungarian female athletes in CSFH, but no significant associations were found among Turkish or Hungarian male athletes. These findings highlight the distinct physical, psychological, and cultural dynamics of combat sports compared to individual and team sports. While social interaction and collective success play a key role in team and racket sports, combat sports prioritize individual performance. This may explain the stronger REA-HGS correlation observed in this discipline. Previous research has identified a strong link between training frequency, duration, and exercise dependence in combat sports (Kostorz et al., 2022), aligning with the positive correlation observed among Hungarian female athletes. Although no significant association was found among Turkish athletes, prior studies have indicated an increased risk of excessive training behaviors, even without significant group differences in exercise dependence (Orhan et al., 2019). To better understand the interplay between exercise dependence and physical strength in combat sports, future research should explore sex-based and cultural influences through comparative analyses.

In the present study, another examined sports discipline was GAS. The analysis revealed a statistically significant positive correlation between REA and HGS among Turkish male athletes whereas no significant relationship was found among Turkish female or Hungarian athletes. The high demands for strength and endurance in gymnastics may contribute to Turkish male athletes’ tendency to develop greater muscular strength, increasing HGS as exercise dependence rises. Additionally, the growing international success of Turkish male gymnasts may further reinforce this association by increasing training intensity and exercise dependence, making the REA-HGS relationship more pronounced. Previous research has highlighted the psychological dimensions of esthetic sports, demonstrating a significant association between dance addiction and mild psychopathology (Maraz et al., 2015). Furthermore, existing literature indicates a high prevalence of eating disorders in sports requiring strict weight management, such as gymnastics, with comorbidity rates reaching up to 40% (Lejoyeux et al., 2008). Further empirical research is required to understand the potential interaction between exercise dependence and physiological performance indicators such as HGS. Such studies could provide deeper insights into the underlying mechanisms forming this relationship.

Although no statistically significant correlations were detected among athlete groups in TSPF, CSFH, LEEBS or PS, the findings are considered valuable data sources for future research. To strengthen statistical validity, the second phase of this study analyzed HGS and REA correlations across country and sex, revealing a significant positive correlation only among Turkish female athletes. These findings indicate that the relationship between HGS and REA is not universal but may vary across specific populations and sex groups. However, the lack of similar studies limits the ability to draw definitive conclusions. This highlights the need for future research to explore the underlying sociocultural and physiological mechanisms influencing this association.

## 5 Strengths and limitations

A key strength of this cross-sectional study is its hard-to-reach cohort of high-level elite athletes across various sports disciplines. The present study enhances scientific knowledge by analyzing a sizeable elite athlete sample concerning the REA and physical performance link. Including data from two countries enables cross-national comparisons, further broadening their scope. By focusing on elite athletes, the study directly addresses a critical part of understanding REA-performance relationships in high-performance sports. Moreover, using standardized and validated measures strengthens methodological rigor, ensuring more reliable findings.

Despite these strengths, several limitations must be acknowledged. First, reliance on a single REA scale may limit the depth of assessment and the robustness of the findings. Second, the exclusive use of HGS as a physical performance measure may restrict generalizability to a broader range of physical measures. Third, the absence of genetic factors, which could influence REA and performance, is another limitation, as their inclusion could have provided more profound insights. Fourth, a lower sample size in specific sports may reduce statistical power, affecting the reliability and interpretability of the results. Fifth, another potential limitation of this study is that HGS may reflect sport-specific adaptations rather than general physical performance, as the relevance of grip strength varies significantly across disciplines. This variability could influence the strength or presence of correlations with exercise addiction risk, especially in sports where upper-limb exertion is minimal. Finally, the cross-sectional research design and volunteer sample limit the inference to causality and the generalizability of the results.

## 6 Conclusion

In conclusion, the present study reveals a relationship between HGS and REA, with significant variations across sports disciplines and sex. In some instances, correlations are high. Turkish female racquet and water sports athletes exhibited a negative correlation, suggesting a healthier exercise balance. In contrast, positive correlations in team and specific sports indicated a stronger relationship between REA and physical strength. These findings align with SDT, emphasizing the role of intrinsic motivation, autonomy, and competence in managing exercise behaviors. The results stress the importance of sport-specific, sex-sensitive approaches in understanding and addressing the REA.

Future empirical research should integrate genetic predisposition (Bulgay et al., 2025), comprehensive physical performance metrics, psychological and neurochemical factors, enhanced sample diversity, and multiple REA assessment tools to deepen the understanding of this complex relationship. Such an approach may enhance the validity and generalizability of the findings across heterogeneous athlete populations, fostering a more holistic framework for studying the interconnection between REA, physiology, and athletic performance.

## Data Availability

The raw data supporting the conclusions of this article will be made available by the authors, without undue reservation.
