# Correction: Association between hand grip strength and exercise addiction: differences by sport category and sex among elite athletes

**DOI:** 10.3389/fpsyg.2025.1755836

**Published:** 2025-12-18

**Authors:** Barış Karaoǧlu, Ebru Ceviz, Şaban Ünver, İsa Çiftçi, Gönül T. Demir, Burcu Güvendi, Celal Bulgay, Merve Alpay, Ferenc Ihasz, Alföldi Zoltan, Rita Kovacsik, Angéla Somogyi, Attila Szabo

**Affiliations:** 1 Sports Science Faculty, Bingol University, Bingol, Türkiye; 2 Yaşar Doǧu Faculty of Sports Science, Ondokuz Mayis University, Samsun, Türkiye; 3 Independent Researcher, Ankara, Türkiye; 4 Sports Science Faculty, Yalova University, Yalova, Türkiye; 5 Physical Education and Sports, Institute of Health Sciences, Sivas Cumhuriyet University, Sivas, Türkiye; 6 Faculty of Health and Sport Sciences, Széchenyi István University, Gyor, Hungary

**Keywords:** athletes, athletic performance, exercise addiction, hand grip strength, sports

The title of this article was erroneously given as: Association between hand grip strength and exercise addiction among high-level athletes: differences by sport category and sex in elite athletes. The correct title of the article is: “Association between hand grip strength and exercise addiction: differences by sport category and sex among elite athletes.”

The original version of this article has been updated.

